# NivobotulinumtoxinA in the Treatment of Glabellar Lines With or Without Concurrent Treatment of Lateral Canthal Lines in Two Phase 3 Clinical Trials

**DOI:** 10.1093/asj/sjae233

**Published:** 2024-11-26

**Authors:** William Coleman, Vince Bertucci, Shannon Humphrey, Joely Kaufman-Janette, Terrence Keaney, David Pariser, Nowell Solish, David Wirta, Robert A Weiss

## Abstract

**Background:**

Botulinum neurotoxins in aesthetic medicine require reconstitution before administration, which may be inconvenient and present errors among injectors.

**Objectives:**

The aim of this study was to evaluate the efficacy and safety of ready-to-use nivobotulinumtoxinA liquid formulation for the treatment of glabellar lines (GL) with or without treatment of lateral canthal lines (LCL).

**Methods:**

Two multicenter, phase 3, double-blind, randomized trials enrolled participants with moderate-to-severe GL (Study 001) or moderate-to-severe GL + LCL (Study 005). Participants received double-blind nivobotulinumtoxinA (20 U) or placebo (Period 1) then ≤2 open-label nivobotulinumtoxinA GL treatments (Period 2) in Study 001 or double-blind nivobotulinumtoxinA 20 U (GL), nivobotulinumtoxinA 44 U (GL + LCL), or placebo (Period 1) then ≤2 double-blind injections of the same treatment (Period 2) in Study 005. The composite primary endpoint was the proportion of participants achieving a ≥2-grade improvement on a facial wrinkle scale at maximum frown on investigator and participant assessment; coprimary endpoints were investigator- and participant-assessed FWS “none or mild” ratings.

**Results:**

At Day 30, significantly higher responder rates were observed for the composite primary endpoint with GL treatment alone (Study 001, 46.1%; Study 005, 45.1%) and GL + LCL (Study 005, 41.3%) vs placebo (0%; all *P* < .001). Responder rates of “none or mild” by investigator and participant assessment, respectively, were significantly higher for GL treatment alone (Study 001, 77.2% and 65.0%; Study 005, 74.3% and 68.8%) and GL + LCL (Study 005, 74.0% and 61.2%) vs placebo (all *P* < .001). Adverse events were similar between treatment groups and placebo.

**Conclusions:**

Liquid nivobotulinumtoxinA was effective and well tolerated for treating moderate-to-severe GL alone or with LCL.

**Level of Evidence: 1 (Therapeutic):**

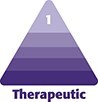

Botulinum neurotoxins (BoNT) are naturally occurring proteins that are a treatment for reducing the appearance of facial wrinkles, including glabellar lines (GL; also known as frown lines) and lateral canthal lines (LCL; also known as crow's feet).^1^ BoNT-A induces temporary chemical denervation and muscle relaxation through cleavage of the synaptosomal-associated protein 25 to prevent acetylcholine release from motor nerve axons, which can diminish the appearance of wrinkles.^1,2^

BoNT-A was first approved by the US Food and Drug Administration (FDA) for the treatment of GL in 2002 and for the treatment of LCL in 2013.^3^ Currently, 6 BoNT-A formulations are approved for aesthetic injection in the USA: onabotulinumtoxinA, daxibotulinumtoxinA, abobotulinumtoxinA, incobotulinumtoxinA, prabotulinumtoxinA, and letibotulinumtoxinA, all of which require reconstitution before administration.^1,4-10^ Managing the supply, storage, and dilution of approved BoNT-A formulations can be complex and prone to errors. Variations in dilution practices among injectors, such as administering different volumes of saline or different types of syringes, can lead to inconsistencies and potential dosage errors due to the lack of standardized procedures.^11^ Ready-to-use liquid formulations can streamline the treatment process, reduce the potential for errors, improve convenience, save time and consumables, and potentially improve patient satisfaction.^12^

NivobotulinumtoxinA (MT10109L; Medytox Inc., Ochang, South Korea) is a 900 kDa botulinum toxin type A complex derived from *Clostridium botulinum* type A strain *Hall* that is isolated through fermentation and purification. Unlike most traditional BoNT-A therapies, nivobotulinumtoxinA offers a ready-to-use liquid formulation with a long shelf-life (up to 36 months), and does not contain human serum albumin as a stabilizer. Instead, it contains L-methionine and polysorbate 20. Studies that report on the concurrent treatment of GL and LCL with a BoNT-A liquid formulation are lacking. Therefore, we investigated the efficacy and safety of nivobotulinumtoxinA in the treatment of moderate-to-severe GL alone (Study 001) and with or without the concurrent treatment of LCL (Study 005) in 2 randomized, placebo-controlled, phase 3 trials.

## METHODS

### Study Design

Studies 001 (NCT03795922; EudraCT 2018-004384-31) and 005 (NCT03721016; EudraCT 2014-005301-21) were global, multicenter, double-blind, randomized, placebo-controlled, parallel-group phase 3 trials of nivobotulinumtoxinA for the treatment of GL alone and GL with or without the concurrent treatment of LCL, respectively. Study 001 was conducted at 14 centers in Belgium, Russia, and the United States from December 2018 to January 2021. Study 005 was conducted at 17 centers in Canada, Germany, the United Kingdom, and the United States from October 2018 to January 2021.

**Figure 1.**
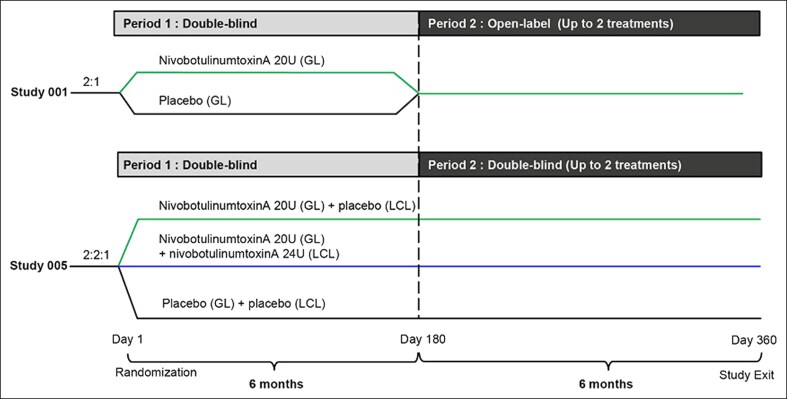
Study design. GL, glabellar lines; LCL, lateral canthal lines.

**Figure 2.**
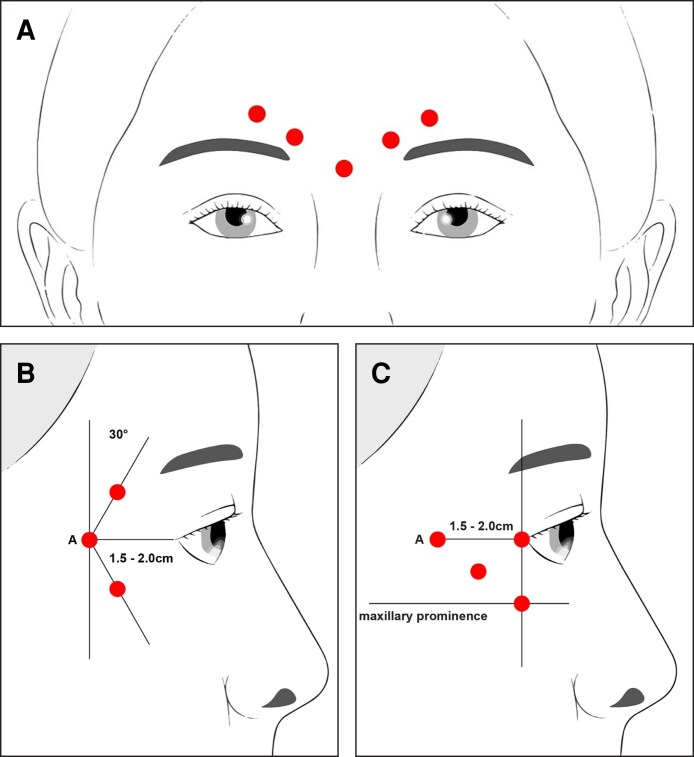
Locations of the 5 glabellar line injections (A). Lateral canthal line injection pattern when lateral canthal lines are distributed above and below the lateral canthus (B); and lateral canthal line injection pattern when lateral canthal lines are primarily distributed below the lateral canthus (C).

Both studies were divided into 2 periods; Period 1 was from Day 1 to Day 180 and Period 2 was from Day 180 to Day 330. In Study 001, participants were randomly assigned 2:1 with an interactive web response system (IWRS) on Day 1 to receive either double-blind nivobotulinumtoxinA 20 units (U) dissolved in sterile solution (L-methionine at 0.2 mg/mL and polysorbate 20 at 0.15 mg/mL) or placebo (all components of the nivobotulinumtoxinA formulation except the toxin) for the treatment of GL during Period 1 (Day 1 to Day 180), with stratification according to the baseline GL severity at maximum frown assessed by the investigator with the facial wrinkle scale (FWS).^13^ Thereafter, all participants received up to 2 open-label treatments with nivobotulinumtoxinA 20 U for the treatment of GL during Period 2 (Day 180 to Day 330) until study completion (Day 360) (Figure 1). The first injection was made in the procerus muscle in the midline. The subsequent 4 injections were made bilaterally (2 injections per side) into each corrugator muscle (Figure 2A). The corrugators were injected inferomedially near the origin of the supratrochlear nerve and superolaterally into the superior middle aspect 1 cm above the bony orbital rim.

In Study 005, participants were randomly assigned 2:2:1 with an IWRS on Day 1 to receive double-blind nivobotulinumtoxinA 20 U dissolved in sterile solution (L-methionine at 0.2 mg/mL and polysorbate 20 at 0.15 mg/mL) for the treatment of GL plus placebo (all components of the nivobotulinumtoxinA formulation except the toxin) for the treatment of LCL; nivobotulinumtoxinA 20 U for the treatment of GL plus nivobotulinumtoxinA 24 U for the treatment of LCL (ie, a total of 44 U nivobotulinumtoxinA for the concurrent treatment of GL and LCL); or placebo (GL)/placebo (LCL) during Period 1 (Day 1 to Day 180), with stratification according to the baseline GL severity at maximum frown, assessed by the investigator with the FWS. Thereafter, all participants received up to 2 double-blind injections of the same treatment during Period 2 (Figure 1). The GL injections were administered as described previously (Figure 2A). The LCL injections were administered as bilaterally symmetrical intramuscular injections at 3 injection sites in the lateral aspect of each orbicularis oculi (Figures 2B, 2C). For both studies, during Period 1, follow-up visits occurred on Days 7 and 14 after injection, followed by monthly visits until study completion (Day 360). During Period 2, follow-up visits occurred 7, 14, 30, and 90 days after each treatment until study completion (Day 360).

The study protocol, all protocol amendments, written study participant information, informed consent form, the investigator's brochure, and any other relevant documents were reviewed and approved by the following independent ethics committees and institutional review boards at each study center. For Study 001, this included the Western Institutional Review Board (Pullyap, WA), Commissie Medische Ethiek (Brussels, Belgium), Local Ethics Committee of FSBEI HE Kazan State Medical University (Kazan, Russia), and Independent Ethics Committee of the City Clinical Hospital (Moscow, Russia). For Study 005, included were the Western Institutional Review Board (Pullyap, WA), Ethics Committee at the Bavarian State Medical Association, BLÄK (Munich, Germany), and East of Scotland Research Ethics Service REC 2 Tayside Medical Science Centre, Ninewells Hospital (Dundee, UK). Both studies were conducted in accordance with the protocol; the ethical principles derived from international guidelines, including the Declaration of Helsinki and Council for International Organizations of Medical Sciences International Ethical Guidelines; applicable International Council for Harmonisation Good Clinical Practice and other guidelines; and applicable laws and regulations.

### Study Participants

Male and female participants aged ≥18 years with bilateral moderate-to-severe GL at maximum frown (assessed by investigator and participant, Study 001 and Study 005) and with moderate-to-severe LCL at maximum smile (assessed by investigator only, Study 005) with the FWS were eligible for enrollment.^13^ Participants with previous exposure to any BoNT subtype, any medical condition that could put the participants at increased risk with exposure to MT10109L, or a medical history or taking medicines that could interfere with treatment evaluation were excluded from these studies.

### Efficacy Assessments

Per FDA requirements, the primary composite endpoint evaluated the proportion of participants who achieved a ≥2-grade improvement from baseline on the FWS according to investigator and participant assessment of GL severity at maximum frown on Day 30 during Period 1 in the intent-to-treat (ITT) population (all randomized participants).^14^ Per European Medicines Agency (EMA) requirements (data on file), the coprimary endpoint evaluated the proportion of participants who achieved a rating of “none or mild” on the FWS according to investigator and participant assessments of GL severity at maximum frown on Day 30 during Period 1 in the modified intent-to-treat (mITT) population (defined as all randomized participants who had a baseline transformed Facial Line Outcomes questionnaire [FLO-11] total score of ≤50).^15^ Key secondary endpoints were the percentage of participants reporting mostly satisfied/very satisfied on the Facial Line Satisfaction Questionnaire (FLSQ) follow-up version, Item 5 for GL at Day 60.^16^ Another key secondary endpoint was the duration of effect, defined by return to moderate or severe FWS rating (equivalent to loss of none or mild rating) for participants who achieved a ≥2-grade improvement from baseline in GL severity at maximum frown according to investigator assessment on Day 30. Key secondary endpoints were assessed in the ITT population.

### Safety Assessments

Safety was assessed in the safety population (all participants who received at least 1 injection of study intervention according to the actual intervention received) as the incidence and severity of treatment emergent adverse events (TEAEs), serious AEs (SAEs), and AEs of special interest.

### Immunogenicity

A 2-stage assay approach was utilized for the detection of binding antibodies against nivobotulinumtoxinA and neutralizing antibodies against nivobotulinumtoxinA in serum. In Stage 1, serum samples were screened for the presence of binding antibodies with a validated enzyme-linked immunosorbent assay in a 3-tier format (screening, confirmation, and determining the titer; Intertek Pharmaceutical Services, Manchester, UK). Serum samples that were positive at screening were subsequently immunodepleted to confirm that the binding antibodies were specific to nivobotulinumtoxinA and then had the titer determined to assess the amount of antibodies present. In Stage 2, samples that were positive in the antidrug antibody confirmatory assay were evaluated in a mouse protection assay (Battelle Memorial Institute, Columbus, OH).

### Statistical Analyses

Per FDA and EMA requirements (data on file), all efficacy analyses were conducted in the ITT and mITT populations, respectively, and safety evaluations were conducted in the safety population.^14^ The level of significance for all statistical tests was 0.05, 2-sided, unless stated otherwise. Missing values for the primary endpoint measures at visits from baseline up to Day 180 were imputed with multiple imputation. Five imputation data sets were generated with the Proc MIANALYZE procedure in SAS software (SAS Institute Inc., Cary, NC).^17^ Specifically, the missing data for age, sex, race, baseline FLO-11 binary indicator (1 if FLO-11 transformed total score ≤50 or 0 if >50) and FWS score at visits were imputed with a multiple imputation method in which each efficacy variable was imputed and stratified by treatment group with the Markov chain Monte Carlo method. The evaluation of the equality of the proportions of responders was based on the Cochran Mantel Haenszel (CMH) test stratified by investigator-assessed baseline GL severity at maximum frown.

## RESULTS

### Baseline Characteristics

**Figure 3.**
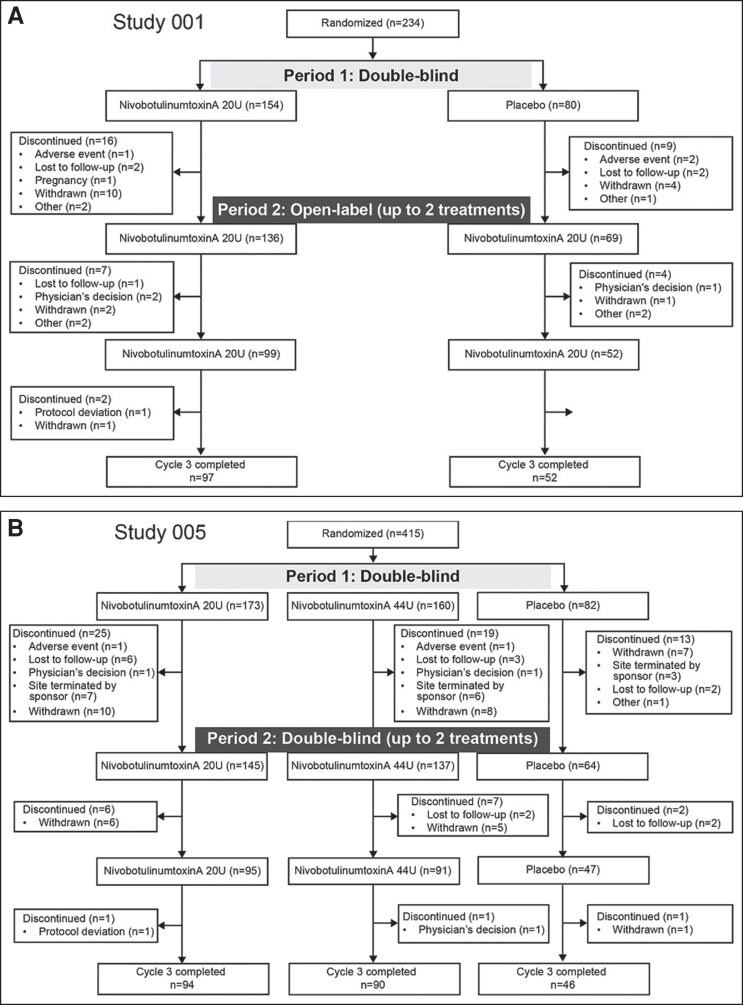
Participant disposition in Study 001 (A) and Study 005 (B).

In Study 001, 234 participants were randomly assigned to receive placebo (*n* = 80) or nivobotulinumtoxinA 20 U for the treatment of GL (*n* = 154) on Day 1 (Figure 3A). In Study 005, 415 participants were randomly assigned to receive placebo (*n* = 82), nivobotulinumtoxinA 20 U for the treatment of GL (*n* = 173), or nivobotulinumtoxinA 44 U for the treatment of GL + LCL (*n* = 160) (Figure 3B). There were no differences in baseline characteristics between treatment groups in both studies (Table 1). Most participants were female (85.5-95.0%) and the mean (standard deviation) age in years was 46.4 (11.6) and 47.3 (12.6) in the placebo and nivobotulinumtoxinA 20 U (GL) groups, respectively in Study 001, and 47.6 (10.9), 47.5 (11.5), and 46.2 (11.2) in the placebo, nivobotulinumtoxinA 20 U (GL), and 44 U (GL + LCL) groups, respectively, in Study 005 (Table 1). At baseline, the proportion of participants with severe GL at maximum frown according to investigator assessment ranged from 54.4% to 59.1%, and the proportion of participants with moderate GL at maximum frown ranged from 40.9% to 45.6%, across the studies (Table 1). In Study 001, the median (range) duration of follow-up was 370.0 days (65.0-522.0 days) in the nivobotulinumtoxinA 20-U group and 369.5 days (8.0-555.0 days) in the placebo group. In Study 005, the median (range) duration of follow-up was 372.0 days (2.0-555.0 days) and 373.0 days (7.0-491.0 days) in the nivobotulinumtoxinA 20-U group and 44-U group, respectively, and 372.0 days (22.0-465.0 days) in the placebo group.

**Table 1.** Baseline Characteristics

	Study 001 *n* = 234		Study 005 *n* = 415		
	Placebo *n* = 80	NivobotulinumtoxinA 20 U (GL) *n* = 154	Placebo *n* = 82	NivobotulinumtoxinA 20 U (GL) *n* = 173	NivobotulinumtoxinA 44 U (GL + LCL) *n* = 160
Age, mean (SD), years	46.4 (11.6)	47.3 (12.6)	47.6 (10.9)	47.5 (11.5)	46.2 (11.2)
Sex, *n* (%)					
Male	4 (5.0)	14 (9.1)	10 (12.2)	25 (14.5)	17 (10.6)
Female	76 (95.0)	140 (90.9)	72 (87.8)	148 (85.5)	143 (89.4)
Investigator FWS GL at maximum frown, *n* (%)					
2 = Moderate	34 (42.5)	63 (40.9)	37 (45.1)	76 (43.9)	73 (45.6)
3 = Severe	46 (57.5)	91 (59.1)	45 (54.9)	97 (56.1)	87 (54.4)
Investigator FWS GL at rest, *n* (%)					
0 = None	4 (5.0)	14 (9.1)	5 (6.1)	12 (6.9)	14 (8.8)
1 = Mild	32 (40.0)	55 (35.7)	34 (41.5)	74 (42.8)	61 (38.1)
2 = Moderate	31 (38.8)	71 (46.1)	30 (36.6)	67 (38.7)	63 (39.4)
3 = Severe	13 (16.3)	14 (9.1)	13 (15.9)	20 (11.6)	22 (13.8)
Participant FWS GL at maximum frown, *n* (%)					
1 = Mild	0	0	1 (1.2)^a^	0	0
2 = Moderate	34 (42.5)	63 (40.9)	36 (43.9)	76 (43.9)	73 (45.6)
3 = Severe	46 (57.5)	91 (59.1)	45 (54.9)	97 (56.1)	87 (54.4)
Participant FWS GL at rest, *n* (%)					
0 = None	5 (6.3)	13 (8.4)	4 (4.9)	9 (5.2)	14 (8.8)
1 = Mild	27 (33.8)	49 (31.8)	30 (36.6)	62 (35.8)	49 (30.6)
2 = Moderate	35 (43.8)	68 (44.2)	35 (42.7)	74 (42.8)	63 (39.4)
3 = Severe	13 (16.3)	24 (15.6)	13 (15.9)	28 (16.2)	34 (21.3)

FWS, facial wrinkle scale; GL, glabellar lines; LCL, lateral canthal lines; SD, standard deviation. ^a^The participant-reported protocol deviation.

### Composite Primary Endpoint

In Study 001, there were significantly more responders (participants with ≥2-grade improvement from baseline in GL severity at maximum frown according to investigator and participant assessment on the FWS) in the nivobotulinumtoxinA 20 U (GL) treatment group (*n* = 71, 46.1%) than in the placebo group (*n* = 0) at Day 30 (*P* < .001) (Table 2). Similarly, in Study 005, there were significantly more responders in the nivobotulinumtoxinA 20 U (GL) treatment group (*n* = 78, 45.1%) and in the nivobotulinumtoxinA 44 U (GL + LCL) treatment group (*n* = 66, 41.3%) than in the placebo group (*n* = 0, *P* < .001) (Table 2).

**Table 2.** Primary Endpoints at Day 30

Composite primary endpoint (ITT population)	Study 001 *n* = 234		Study 005 *n* = 415		
	Placebo *n* = 80	NivobotulinumtoxinA 20 U (GL) *n* = 154	Placebo *n* = 82	NivobotulinumtoxinA 20 U (GL) *n* = 173	NivobotulinumtoxinA 44 U (GL + LCL) *n* = 160
Responder, *n* (%)	0	71 (46.1)	0	78 (45.1)	66 (41.3)
*P* value	—	<.001	—	<.001	<.001
Coprimary endpoint (mITT population)	Placebo *n* = 68	NivobotulinumtoxinA 20 U (GL) *n* = 123	Placebo *n* = 67	NivobotulinumtoxinA 20 U (GL) *n* = 155	NivobotulinumtoxinA 44 U (GL + LCL) *n* = 134
Investigator FWS					
Responder, *n* (%)	3 (4.4)	95 (77.2)	3 (4.5)	115 (74.2)	99 (73.9)
*P* value		<.001		<.001	<.001
Participant FWS					
Responder, *n* (%)	3 (4.4)	80 (65.0)	2 (3.0)	107 (69.0)	82 (61.2)
*P* value		<.001		<.001	<.001

The composite primary endpoint was the proportion of participants achieving a ≥2-grade improvement in FWS from baseline according to investigator and participant assessment of GL severity at maximum frown in the ITT population at Day 30. The coprimary endpoint was the proportion of participants who had GL severity at maximum smile of “none or mild” based on investigator and participant FWS ratings in the mITT population at Day 30. FWS, facial wrinkle scale; GL, glabellar lines; ITT, intention-to-treat; LCL, lateral canthal lines; mITT, modified intention-to-treat.

### Coprimary Endpoints

In Study 001, the proportion of participants who had GL severity of “none or mild” at maximum frown according to investigator assessment of FWS was significantly higher following the first treatment with nivobotulinumtoxinA 20 U (*n* = 95, 77.2%) than with placebo (*n* = 3, 4.4%) at Day 30 (*P* < .001) (Table 2). The proportion of participants who had GL severity of “none or mild” at maximum frown according to participant assessment of FWS was also significantly higher following the first treatment with nivobotulinumtoxinA 20 U (*n* = 80, 65.0%) than with placebo (*n* = 3, 4.4%) at Day 30 (*P* < .001) (Table 2).

**Figure 4.**
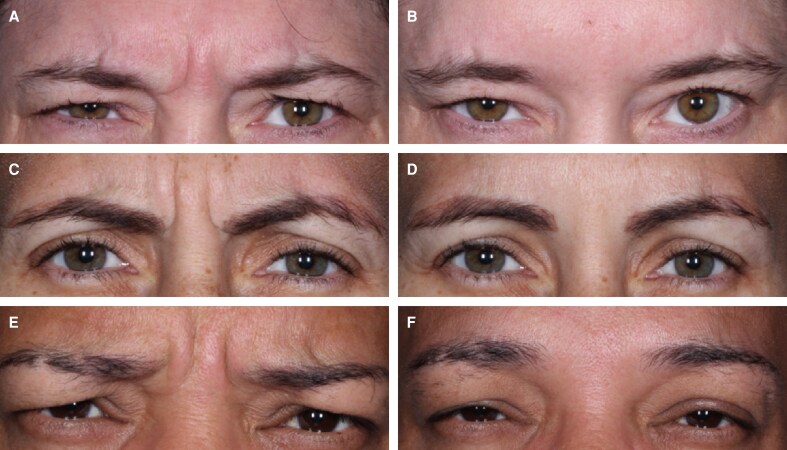
Representative before-and-after patient photographs from 3 patients (A-F) included in this study at baseline (A, C, E) and Day 30 (B, D, F) at maximum frown. (A, B) 46-year-old female, (C, D) 41-year-old female, (E, F) 40-year-old male.

In Study 005, the proportion of participants who had GL severity of “none or mild” at maximum frown according to investigator assessment of FWS was significantly higher following the first treatment with nivobotulinumtoxinA 20 U (GL) (*n* = 115, 74.2%) and nivobotulinumtoxinA 44 U (GL + LCL) (*n* = 99, 73.9%) than with placebo (*n* = 3, 4.5%) at Day 30 (both *P* < .001) (Table 2). The proportion of participants who had GL severity of “none or mild” at maximum frown per participant FWS was also significantly higher following the first treatment with nivobotulinumtoxinA 20 U (GL) (*n* = 107, 68.8%) and nivobotulinumtoxinA 44 U (GL + LCL) (*n* = 82, 61.2%) than with placebo (*n* = 2, 3.0%) at Day 30 (both *P* < .001) (Table 2). Representative photographs of patients before and after treatment at maximum frown are shown in Figure 4.

### Secondary Endpoints

**Figure 5.**
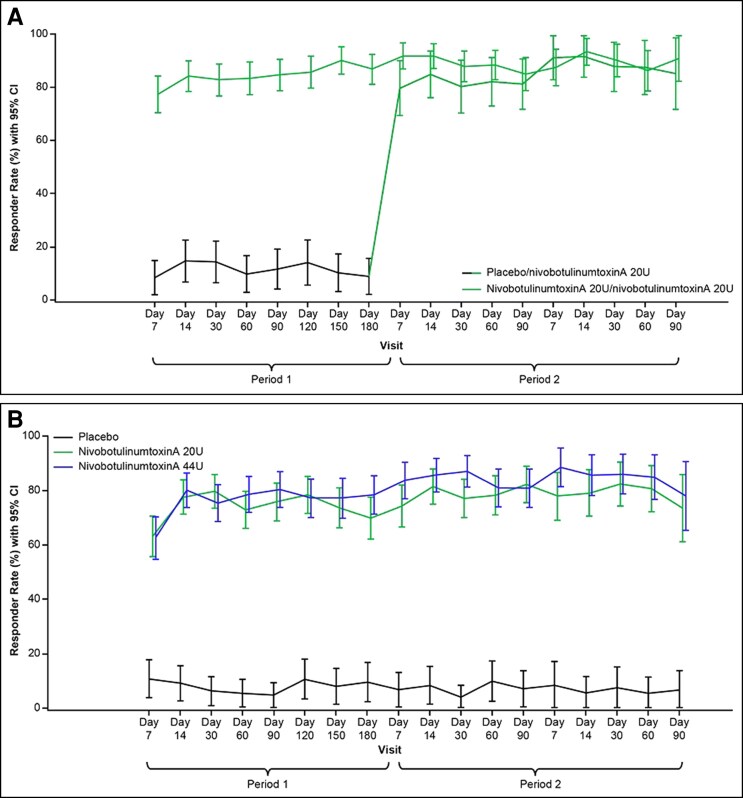
Proportion of participants in Study 001 (A) and Study 005 (B) who reported “mostly satisfied” or “very satisfied” on FLSQ. Based on the FLSQ follow-up version, Item 5 (ITT population). CI, confidence interval; FLSQ, Facial Line Satisfaction Questionnaire; ITT, intention-to-treat.

In Study 001, the proportion of participants who reported mostly satisfied/very satisfied, according to assessment on the FLSQ follow-up version, Item 5, at Day 60, was significantly higher in the nivobotulinumtoxinA 20 U (GL) group (*n* = 121, 83.4%) than in the placebo group (*n* = 7, 9.7%, *P* < .001). Participant satisfaction was similar in both groups during Period 2 in which they received up to 2 open-label nivobotulinumtoxinA 20 U (GL) treatments (Figure 5A). A similar finding was observed in Study 005 (Figure 5B) in which the proportion of participants who reported mostly satisfied/very satisfied according to assessment on the FLSQ follow-up version, Item 5, at Day 60 was significantly higher in the nivobotulinumtoxinA 20 U (GL) group (*n* = 116, 73.0%, *P* < .001) and in the nivobotulinumtoxinA 44 U (GL + LCL) group (*n* = 118, 78.7%, *P* < .001) than in the placebo group (*n* = 4, 5.4%). There were consistently more responders in the nivobotulinumtoxinA 20-U (GL) and 44-U (GL + LCL) groups vs the placebo group throughout the study (Figure 5B).

**Figure 6.**
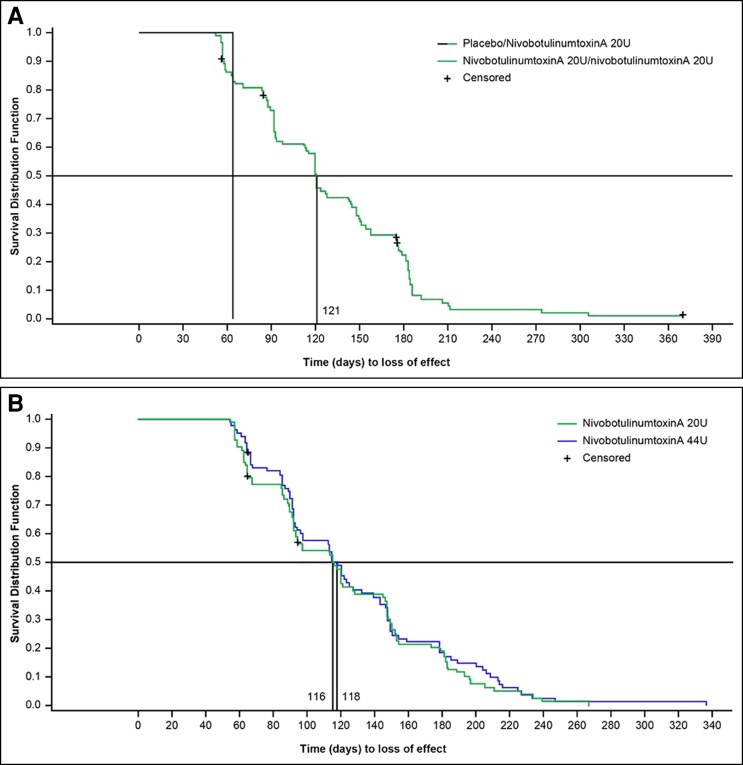
Median duration before returning to moderate or severe, based on investigator GL FWS at maximum frown utilizing the Kaplan-Meier method in Study 001 (A) and Study 005 (B). The duration of GL study intervention effect was defined as the time to loss of treatment effect (return to moderate or severe GL at maximum frown) in the proportion of patients who achieved a ≥2-grade improvement from baseline in GL severity at maximum frown at Day 30 of Period 1 according to investigator assessments with the FWS. Responder who reached end of treatment cycle or exited the study before becoming a nonresponder is considered censored. FWS, facial wrinkle scale; GL, glabellar lines.

In Study 001, the median time to loss of treatment effect (return to moderate or severe GL severity at maximum frown on the FWS) was 121.0 days (17.3 weeks) in the nivobotulinumtoxinA 20 U (GL) group (Figure 6A). In Study 005, the median time to loss of treatment effect was 116.0 days (16.6 weeks) in the nivobotulinumtoxinA 20 U (GL) group and 118.0 days (16.9 weeks) in the nivobotulinumtoxinA 44 U (GL + LCL) group (Figure 6B).

### Safety

In Study 001, 35 (43.8%) participants in the placebo group and 118 (52.9%) participants in the nivobotulinumtoxinA 20 U (GL) group experienced at least 1 TEAE (Table 3). In Study 005, 31, 82, and 61 participants (37.8%, 47.1%, and 38.4%) in the placebo, nivobotulinumtoxinA 20 U (GL), and nivobotulinumtoxinA 44 U (GL + LCL) treatment groups, respectively, experienced at least 1 TEAE (Table 3).

**Table 3.** Incidence and Frequency of Treatment-emergent Adverse Events

	Study 001		Study 005		
	Placebo *n* = 80	NivobotulinumtoxinA 20 U (GL) *n* = 223	Placebo *n* = 82	NivobotulinumtoxinA 20 U (GL) *n* = 174	NivobotulinumtoxinA 44 U (GL + LCL) *n* = 159
Participants with ≥1 TEAE, *n* (%)	35 (43.8)	118 (52.9)	31 (37.8)	82 (47.1)	61 (38.4)
Headache	4 (5.0)	22 (9.9)	4 (4.9)	14 (8.0)	6 (3.8)
Injection site pain	4 (5.0)	16 (7.2)	4 (4.9)	9 (5.2)	7 (4.4)
Eyelid ptosis	0	3 (1.3)	0	2 (1.1)	1 (0.6)
Serious AE, *n* (%)	3 (3.8)	5 (2.2)	2 (2.4)	3 (1.7)	5 (3.1)
Treatment related	0	0	0	0	0
Death, *n* (%)	0	0	0	0	0

TEAEs were reported in ≥5% of participants in any treatment group (safety population). AE, adverse event; GL, glabellar lines; LCL, lateral canthal lines; TEAE, treatment-emergent adverse event.

In both studies, the most common TEAEs (those reported in ≥5% of participants) were headache (Study 001, 9.9% in the nivobotulinumtoxinA 20 U group and 5.0% in the placebo group; Study 005, 8.0% in the nivobotulinumtoxinA 20 U [GL] group, 3.8% in the 44 U [GL + LCL] group, and 4.9% in the placebo group) and injection-site pain (Study 001, 7.2% in the nivobotulinumtoxinA 20 U group and 5.0% in the placebo group; Study 005, 5.2% in the nivobotulinumtoxinA 20 U [GL] group, 4.4% in the 44 U [GL + LCL] group, and 4.9% in the placebo group) (Table 3). Eyelid ptosis was reported in only 3 participants during each study; in Study 001, 3 participants (1.3%) in the nivobotulinumtoxinA 20 U treatment group reported an eyelid ptosis, and in Study 005, 2 participants (1.1%) in the nivobotulinumtoxinA 20 U (GL) group and 1 participant (0.6%) in the nivobotulinumtoxinA 44 U (GL + LCL) group reported an eyelid ptosis (Table 3). There were no treatment-related SAEs or deaths in either study.

### Immunogenicity

Throughout Study 001, 1 participant who received placebo during Period 1 followed by 2 nivobotulinumtoxinA 20 U treatments during Period 2 had a positive result for the binding antibody test before the first nivobotulinumtoxinA treatment (Cycle 2 Day 1); antibody titers remained low (≤30) through study exit. This participant had a positive neutralizing antibody result after the nivobotulinumtoxinA treatment; however, neutralizing antibody results before the first nivobotulinumtoxinA treatment were not obtained due to insufficient sample volume. This participant showed transient ≥2-grade improvement in treatment response with the first treatment at Day 14, but did not achieve treatment response at any subsequent visits. The participant did not report any TEAEs throughout the study. In Study 005, 1 participant in the nivobotulinumtoxinA 44 U treatment group (GL + LCL) who had negative binding and neutralizing antibody results at Day 1 had positive results for binding antibody and neutralizing antibody at Day 90 during Period 1, with very low antidrug antibody titers (<10). Despite this, the participant showed ≥2-grade improvement in both investigator- and participant-rated FWS GL scores. The participant had negative results for binding and neutralizing antibody tests at all subsequent visits, including study exit, and did not report any TEAEs throughout the study.

## DISCUSSION

Treatment of GL alone with nivobotulinumtoxinA 20 U or with the concurrent treatment of LCL with the novel liquid formulation nivobotulinumtoxinA 24 U over 3 treatment cycles was efficacious and well tolerated in these 2 clinical trials. Significant improvements were observed with nivobotulinumtoxinA treatment for GL alone or GL plus LCL compared with placebo for the primary endpoints. For the composite primary endpoint, the proportion of responders who achieved ≥2-grade improvement was significantly higher than placebo for the treatment of GL and for GL with concurrent treatment of LCL. For the coprimary endpoint, significantly higher responder rates were also observed in the nivobotulinumtoxinA group (*P* < .001 for each dose of nivobotulinumtoxinA vs placebo). NivobotulinumtoxinA was well tolerated and there were no unexpected safety findings for a BoNT-A therapy.

The US-approved BoNT-A therapies for the treatment of GL and GL and/or LCL all require reconstitution before administration, which can be inconvenient and may introduce errors and inconsistencies among injectors.^5-10^ Some injectors opt for larger reconstitution volumes to ensure broader diffusion in specific areas, whereas smaller volumes may be chosen for precise BoNT-A administration; therefore, in practices with multiple injectors utilizing varied reconstitution volumes and syringe types, confusion and potential dosage errors can arise.^11,18^ There is an unmet need for liquid formulations, which are easier to store and use in practice and are associated with a lower risk of administration errors.^5-10^ Although other liquid formulations of BoNT-A are under investigation, such as (liquid) abobotulinumtoxinA and relabotulinumtoxinA for the treatment of GL, there are no approved BoNT-A liquid formulations for the concurrent treatment of GL and LCL in the USA.^12,19,20^

Although direct comparisons between our study and previous studies cannot be made because of differences in patient populations, study designs, and endpoints assessed, our results for nivobotulinumtoxinA showed significant improvement in investigator- and participant-rated outcomes, which was consistent with approved BoNT-A therapies requiring reconstitution before administration for the treatment of GL. At least a 2-grade improvement in composite investigator- and participant-rated outcomes has been reported in studies of the formulations of incobotulinumtoxinA (20 U), daxibotulinumtoxinA (40 U), prabotulinumtoxinA (20 U), and onabotulinumtoxinA (20 U), which require reconstitution before treatment of GL.^21-24^ Consistent with these previous studies, we showed that there were significantly more responders vs placebo for the primary endpoint of ≥2-grade improvement in FWS according to investigator- and participant-rated assessment on the FWS with liquid nivobotulinumtoxinA treatment of GL alone, at rates of 46.1% and 45.1% in Study 001 and Study 005, respectively. Furthermore, we showed a consistent effect for the treatment of GL with concurrent treatment of LCL, at a rate of 41.3%. Regarding the proportion of responders who reported a “none or mild” rating, significantly more investigator-rated and participant-rated responders were observed in the BoNT-A treatment group vs placebo for the treatment of GL in studies reporting on liquid abobotulinumtoxinA at day 29 or 30.^12,19^ Similarly, the proportion of participants who achieved a rating of “none or mild” ranged from 74.3% to 77.2% by investigator assessment and from 61.2% to 68.8% by participant assessment in our study.

In previous studies of other BoNT-A therapies, similar rates of TEAEs were reported between participants who received BoNT-A treatment and those who received placebo, with the most frequently reported TEAEs being injection-site pain and headache, and eyelid ptosis reported at rates ranging from 1.6% to 3.0%.^12,19,22,23,25^ Our studies were similar in that headache and injection-site pain were the most commonly reported TEAEs and eyelid ptosis was reported at a rate of 0.6% to 1.3%. The incidence of headache was shown to decrease after more than 1 treatment cycle in our studies.

Across the clinical trial program of liquid nivobotulinumtoxinA, including the studies reported here and trials of nivobotulinumtoxinA for the treatment of LCL alone or with concurrent treatment of GL (Carruthers et al 2024 in development), very few participants reported treatment-induced neutralizing antibodies. In these studies, neutralizing antibody was detected in 2 participants; although, the interpretation of these positive results was limited because of sample volume insufficiency in 1 participant and lack of correlation with clinical response in the other. The mouse protection assay is the gold standard technique for detecting neutralizing antibody. However, it still has some limitations, including the lack of a universally accepted threshold for positive detection, and limited consideration for the dynamics of BoNT-A antibody formation.^26^ Therefore, interpretation of BoNT-A antibody testing results is challenging.

A limitation of our studies was that most patients were female; however, this is consistent with studies of other BoNT-A therapies.^19,21-23,25^ A strength of our studies was that they included a robust composite primary endpoint that incorporated the investigator and participant rating of improvement of at least 2 grades on the FWS in the assessment of GL.

## CONCLUSIONS

In conclusion, the results of these two phase 3 clinical trials support the efficacy and safety of the liquid formulation of nivobotulinumtoxinA in the management of moderate-to-severe GL with or without concurrent treatment of LCL.
